# Prevalence of intestinal parasites among patients of a Ghanaian psychiatry hospital

**DOI:** 10.1186/s13104-015-1634-6

**Published:** 2015-11-05

**Authors:** Kwabena O. Duedu, Yaw A. Karikari, Simon K. Attah, Patrick F. Ayeh-Kumi

**Affiliations:** Department of Medical Microbiology, School of Biomedical and Allied Health Sciences, University of Ghana, Accra, Ghana; Department of Medical Laboratory Sciences, School of Biomedical and Allied Health Sciences, University of Ghana, Accra, Ghana; Department of Biomedical Sciences, University of Health and Allied Sciences, Ho, Ghana

**Keywords:** Parasitic infections, Tropics, Psychiatric patients, Ghana, Sub-Saharan Africa

## Abstract

**Background:**

Neglected tropical diseases are of major concern to sub-Saharan African countries. Though efforts to monitor the prevalence and control are in place, these are mostly restricted to groups within the population. This study was performed to determine the prevalence among patients of a Ghanaian psychiatric hospital and find out whether there is a reason for active monitoring in this population.

**Methods:**

A cross-sectional study was performed to determine the prevalence of intestinal parasites among patients of a Ghanaian psychiatric hospital. Stool samples were collected and analyzed in addition to data.

**Results:**

Of the 111 patients studied, asymptomatic carriage of parasites was 13.5 % and was higher in males (18.8 %) than in females (4.8 %). Carriage of parasites decreased with age but increase with duration of admission.

**Conclusion:**

This is the first report of parasitic pathogens among patients of a psychiatric institution in Ghana. The data shows that there are risks of transmission of infectious diseases via the oral route hence, the need for regular monitoring and intervention is emphasized.

## Background

Parasitic diseases are common in poor countries and accounts for approximately 200,000 deaths per year [1–4]. Their occurrence is often associated with socioeconomic and environmental factors. Overcrowding, limited access to clean water and poor personal hygiene are frequently associated with intestinal parasitic infections [2]. Intestinal parasitic infections have detrimental effects on the survival, appetite, growth, school attendance and cognitive performance of infected individuals [5].

High prevalence of intestinal parasitic infections has been reported among psychiatric facilities [6–8]. However, none of these studies are related to psychiatric hospitals in Ghana. Additionally, there is not current data on the epidemiology of mental disorders in Ghana making it difficult to estimate burden [9]. It is assumed based on the World Health Organization (WHO) epidemiological formula for estimation of psychiatric problems in a given population and Ghana’s 2010 census results that about 240,000 people suffer from severe mental disorders [10]. Despite this high burden, the number of health staff taking care of them disproportionate. There’re only 18 psychiatrists and 1200 nurses working in the mental health field in Ghana [11]. Ghana has recently passed a mental health bill aimed at strengthening and improving mental well-being.

Public hospitals Ghana are often overcrowded with some patients being forced to lie on benches and mats in the out patient’s departments (OPDs), corridors and other areas whilst receiving treatment. The overcrowding situation is much worse in the few psychiatric hospitals around. The characteristics of patients in psychiatric facilities are often different from those in acute medical facilities [12]. They are mobile and mingle freely on the wards. Overcrowding in health facilities is often associated with outbreaks of nosocomial infections. To control this, the Society for Healthcare Epidemiology of America/Association for Professionals in Infection Control and Epidemiology has provided guidelines on infection prevention and control in long-term care facilities with sections covering psychiatric facilities [13, 14]. The recommendations are however difficult to implement in psychiatric facilities. This is because, psychiatric patients may not cooperate with the hygienic measures among others [12]. It is therefore important to maintain active surveillance of the prevalence and incidence infectious pathogens in psychiatric institutions to help determine when to introduce specific interventions such as immunizations. In order to obtain preliminary data on the prevalence of infectious diseases among patients of psychiatric facilities in Ghana, we set out to investigate carriage of parasitic pathogens at one of the three psychiatric hospitals in the country.

## Methods

### Study design and setting

A cross-sectional study was conducted in the Accra Psychiatric Hospital which has a population of about 850 patients. The study was conducted between May and August 2012. The wards had no hand washing facilities except the nurses’ stations.

### Participants and variables

Patients that were on admission in the hospital were included in this study. Patients on anti-parasitic drugs or antibiotics were excluded from the study. Simple random sampling was used to recruit study participants that met the inclusion criteria. Written consent was sought from caregivers on behalf of the patients who were incapable of consenting. Demographic data such as age, gender, duration on admission and ward were collected using a case data form. Wide neck leak proof containers were pre-labelled with name, age, sex and ward and given to participants or caregivers to produce stool samples.

### Data sources/management

Stool samples were examined macroscopically for colour, consistency, presence of blood, mucus, pus and large worms [15]. This procedure forms part of routine examination of stool in Ghanaian medical laboratories and was done to obtain clues as to what infections might be present. Infections with whipworms and hookworms for example will cause bleeding and this will lead to blood found in the stool. Additionally, tapeworm proglottids were also being looked out for. Consistency of the stool was also checked to determine whether there were any diarrheic stool or stool with unusual consistency. Stool samples were then fixed with 10 % formalin and transported to the study laboratory on ice.

The parasites were examined by direct wet mount and formol-ether concentration [15]. A portion of stool was examined directly (unstained and stained with iodine) and another portion concentrated. Four slides were prepared for each concentrated sample. Two of the slides were prepared and observed directly unstained and stained with iodine. The other two slides were stained with the modified Ziehl–Neelsen stain [15] for the detection of coccidian parasites. Slides were observed with both low and high (oil) magnifications.

### Study size

In order to achieve a 95 % confidence level, the minimum study size was determined using the formula n = (z^2^ × p(1 − p))/e^2^ where n is the sample size, z is the standard score of 95 % confidence interval, p is the prevalence (since no previous data existed, 50 % was used) and e is the margin of error (1.96) with significance level set at p = 0.05.

### Data handling and statistical analysis

The data was entered into Microsoft Excel and analysed with IBM SPSS v21 (IBM Corporation). Differences between the prevalence in males and females were tested using Z-test for proportions. Proportions of infected and non-infected across age-groups or duration of stay were compared using the Pearson Chi Square test. The differences were considered significant when the *p* value obtained was less than 0.05.

### Ethical approval

The study was approved by the Ethics Committee of the School of Allied Health Sciences. Ethics Identification Number: SAHS-ET/10359980/AA/26A/2012–2013. Permission was sought from management of the hospital. Participation was voluntary and written consent was taken in accordance with the ethical committee’s guidelines. Consent for patients in no mental or physical capacity to do so was obtained from caregivers or relatives.

## Results

**Fig. 1:**
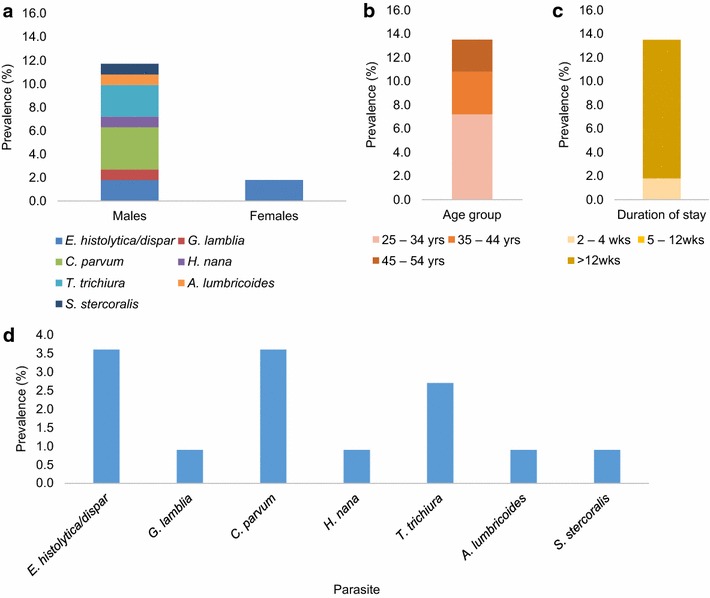
Prevalence of parasites according to gender, age-group and duration of stay at the hospital. The contribution of each group/category to the overall prevalence (13.5 %) is shown as stacked bars for gender (**a**), age-group (**b**) and duration of stay (**c**). The contribution of each parasite to the overall prevalence is given in (**d**). *E. histolytica/dispar* and *C. parvum* were the most prevalent with each being 3.6 %. *E. histolytica/dispar* was the only parasite isolated from females. No infection was recorded for the patients who were more than 54 years old or had been at the facility for 5–12 weeks

A total of 111 patients aged 25–60 years provided adequate stool samples for the study; 69 (62.2 %) were males (mean age 40.58 years) and 42 (37.8 %) were females (mean age 38.0 years). Of the total samples examined 15 were positive for intestinal parasites (13 male and 2 female samples). Positive results is the accumulation of direct wet mount, concentration and ZN techniques. The prevalence of intestinal parasites was 13.5 % with gender specific prevalence being 18.8 and 4.8 % in males and females respectively. There was a significant difference between the prevalence among males and females (*p* = 0.036, Z = 2.104). Seven different types of parasites were identified. These included *Entamoeba histolytica/dispar* cysts, *Giardia lamblia* trophozoites, *Cryptosporidium parvumoocysts*, *Hymenolepis nana* ova, *Trichuris trichiura* ova, *Ascarislumbricoides* ova, and *Strongyloides stercoralis* larvae. The distribution of these parasites is given in Fig. 1. *E. histolytica* and *C. parvum* were the most prevalent protozoan parasites whereas *T. trichiura* was the most prevalent worm.

Of the infected individuals, only two were females. Both females were infected with *E. histolytica/dispar*. The two did not come from the same ward. We also found out that patients with >12 weeks stay were more likely infected. Intestinal parasitic infection was more frequent in younger patients (25–34 years) than the older patients (Fig. 1). There was however no significant difference between the proportions of infection between the various age-groups (*p* = 0.462) or the duration of stay (*p* = 0.284).

## Discussion

Infectious diseases particularly tropical diseases such as intestinal parasitosis are associated with the poor. As a result, there are various efforts by organizations such as the World Health Organization (WHO) and governments to understand the epidemiology as well as provide interventions such as treatment and prevention. The WHO Parasitic Diseases Task Force identified and prioritized parasites that could be transmitted by food to humans as well as produce a substantive burden of disease [16]. In this study, we found high prevalence of *E. histolytica/dispar* and *C. parvum* infections which were also found by the taskforce to have second and third highest global prevalence after *G. lamblia*. Of these, *C. parvum* has the highest prevalence in Africa and it is not surprising to find it to be one of the most prevalent within our study population. *C. parvum* and *E. histolytica* have long been reported to be common causes of diarrhea in Ghana [17–22]. The available data are however skewed to children, AIDS patients and pregnant women. There is currently no data on prevalence of infectious diseases among patients of psychiatric institutions in Ghana.

The species of parasites we identified are related by their modes of transmission. All except *S. stercoralis* get into humans via the mouth. Identification of these parasites suggests that food and water might have been the sources of infection. Food and water in Ghana have been associated with parasitic organisms. We recently reported prevalence rates of parasites associated with vegetables in Ghana [23, 24]. Other reports of parasitic as well as other microbial pathogens association with water have also been published [25, 26]. Studies on intestinal parasites on people in institutionalized facilities in Ghana are not common. In a study conducted among inmates of a Ghanaian orphanage, we found similar prevalence rates of intestinal parasites [27]. Although these have not been linked directly to parasitic infections in the population, their presence in both humans and the food we eat suggests a strong association [28]. *S. stercoralis* on the other hand is transmitted to humans via skin penetration. Patients of the hospital are often confined to their wards. This however does not rule out any possibility of transmission although the risk is very low. With only one person found to have this infection, the probable explanation could be an infection that was brought into the facility. The single infection of strongiloidiasis was from a patient who had been in the facility for less than 2 weeks.

Overall the presence of intestinal parasites suggests that, there could be other potentially pathogenic infections among the patients. Although proportionally the number of males in this study was also higher, the prevalence based on gender suggests males are more likely infected than females. Overcrowding which is common in the facility could cause rapid spread and outbreaks of diseases if interventions to prevent transmission are not put in place. The need to include patients of the facility in surveillance programmes as well as deworming exercises is strongly encouraged. Reports of infection was handed to the management of the hospital for treatment to be given to the patients.

## Conclusions

Although there was high prevalence of intestinal parasites among patients there is no evidence whether inmates acquired the infections within the facility or prior to admission. Although the infections were not associated with any direct clinical signs (e.g. diarrhoea) they are of medical and public health importance. Active surveillance could reveal whether the infections are acquired within the facility or prior to admission. Transmission of these parasites within the facility could be a marker for accessing the hygienic conditions in the facility. Regular deworming and check-ups as well as diagnosis and treatment upon arrival is recommended.

## Limitations

The sample size is small and represents just about 13 % of the total population of the facility. The study is the first of its kind in the facility and we were restricted by management to this population size. A bigger survey which will incorporate treatment and other variables is planned pending appropriate funding. Access to medical history was limited, hence, our inability to link our findings to any clinical conditions. Other limitations include lack of baseline data when patients arrive at the facility. We did not follow-up to determine whether treatment had been administered and whether it had been effective. The detections methods are not perfectly sensitive. Likewise, there was no specificity of differentiating *E. histolytica* from *E. dispar*.

## Abbreviations

WHO: World Health Organization
AIDS: acquired immune deficiency syndrome
OPD: out patient’s department
